# Prevalence and Determinants of Painful and Painless Neuropathy in Type 1 Diabetes Mellitus

**DOI:** 10.3389/fendo.2019.00402

**Published:** 2019-06-28

**Authors:** Margarida Barbosa, Ana Saavedra, Sofia Oliveira, Ligia Reis, Filipa Rodrigues, Milton Severo, Reinhard Sittl, Christoph Maier, Davide M. Carvalho

**Affiliations:** ^1^Department of Anesthesiology, Centro Hospitalar Universitário São João, Porto, Portugal; ^2^Faculty of Medicine, Universidade do Porto, Porto, Portugal; ^3^Instituto de Investigação e Inovação em Saúde, Universidade do Porto, Porto, Portugal; ^4^Department of Endocrinology, Diabetes and Metabolism, Centro Hospitalar Universitário São João, Porto, Portugal; ^5^Department of Anesthesiology of Hospital Espirito Santo, Évora, Portugal; ^6^Department of Anesthesiology, Hospital do Divino Espírito Santo, Ponta Delgada, Portugal; ^7^Epidemiology Research Unit, Public Health Institute, Universidade do Porto, Porto, Portugal; ^8^Hautklinik, Universitätsklinikum Erlangen, Erlangen, Germany; ^9^Department of Pain Medicine, BG University Hospital Bergmannsheil GmbH, Bochum, Germany

**Keywords:** type 1 diabetes, neuropathy, painless, painful, determinants/risk factors

## Abstract

**Aims:** To evaluate (1) the prevalence of diabetic distal symmetrical sensory–motor polyneuropathy (DSPN) and painful DSPN among patients with type 1 diabetes mellitus (DM1) aged over 18 years and (2) the determinant factors of neuropathy and pain in those patients.

**Materials and Methods:** An epidemiological, cross-sectional, observational study was performed; 330,386 people were included, and a total of 444 people were diagnosed with DM1. After exclusion of possible confounders, 360 patients were assessed for painless and painful DSPNs using neurological examination and questionnaires for neuropathy and pain. Odds ratio (OR) and confidence intervals (95% CI) were estimated using multinomial logistic regression models. The analysis was based on a framework with four conceptual levels that consider feasible pathways between several risk factors: (1) socio-demographic factors and diabetes duration, (2) patient habits, (3) co-morbidities, and (4) metabolic factors and disease complications.

**Results:** The prevalence of DSPN and painful DSPN were 42.8 and 18.9%, respectively. Diabetes duration was positively associated with painful (OR = 1.107, 95% CI: 1.107–1.139) and painless DSPN (OR = 1.069, 95% CI: 1.043–1.096). Education level was negatively associated with painful DSPN (OR = 0.889, 95% CI: 0.826–0.957). Sex (female) was positively associated only with painless DSPN (OR = 1.769, 95% CI: 1.007–3.107). Being a current or former smoker was positively associated only with painless DSPN (OR = 1.940, 95% CI: 1.069–3.518). Hypertension was positively associated with painful DSPN (OR = 2.474, 95% CI: 1.110–5.512) and painless DSPN (OR = 2.565, 95% CI: 1.252–5.256). Glycated hemoglobin (HbA1c) was positively associated only with painless DSPN (OR = 1.193, 95% CI: 1.018–1.399).

**Conclusions:** Diabetes duration and hypertension have a direct impact on the development of painful and painless DSPN. However, female sex and HbA1c have a direct effect only on the development of painless DSPN, and education level has an indirect effect on the development of painful DSPN. Therefore, it can be concluded that different etiological factors have different contributions to the development of neuropathy and pain.

## Introduction

Diabetes mellitus (DM) is a metabolic disorder associated with micro- and macro-vascular complications. DM is a major cause of non-traumatic amputations. It has a major impact on quality of life, and represents a serious economic burden on the health system (1). Diabetic distal symmetrical sensory-motor polyneuropathy or distal symmetrical polyneuropathy (DSPN), which is the most common complication of DM, accounts for 75% of diabetic neuropathies (2, 3). The Toronto Consensus Panel on Diabetic Neuropathy defines DSPN as a “symmetrical, length-dependent sensorimotor polyneuropathy, attributable to metabolic, and microvessel alterations, as a result of chronic hyperglycemia exposure (diabetes) and cardiovascular risk covariates” (4, 5). Among diabetic patients, this neuropathy has a typical “glove and stocking” distribution pattern. Worldwide, the incidence and prevalence of DSPN vary greatly, but evidence from several large observational cohorts (6) such as the Diabetes Control and Complications Trial (DCCT) and Epidemiology of Diabetes Interventions and Complications (EDIC) (7) suggest that DSPN occurs in at least 20% of people with type 1 DM (DM1) after 20 years of disease duration (8). The prevalence of DSPN among patients with DM1 is known to differ from that of patients with type 2 diabetes; Young's study found a 22.7% (21.0–24.4 %) prevalence in patients with DM1 (9), with duration of diabetes as the factor with the highest association with DSPN. The risk factors for the development of DSPN in patients with DM1 are age, duration of diabetes, poor glycemic control (5, 10), height (11) (may be because nerve length is bigger in taller persons), smoking (12), hypertension, and lipid profile (13, 14). Some studies have also found a strong association between DSPN and cardiovascular autonomic neuropathy, hypertension, nephropathy, and retinopathy (15). A weak association with peripheral arterial disease, cardiovascular disease, and depression was found. Older patients with diabetes and DSPN, specially those who are insulin treated, have worse performance on walking tests, which is due to proprioception affection, than those without DSPN (16). In the Rochester Diabetic Neuropathy Study (RDNS) (17), mean glycated hemoglobin (HbA1c), diabetic retinopathy, increased albumin excretion rate (AER), and diabetes duration were found to be the major risk factors for severity of DSPN after 7 years of follow-up. Thus, risk factors for neuropathy include smoking, obesity, older age, lipid profile characteristics, longer diabetes duration, and increased diastolic blood pressure (18–20). DSPN is also associated with substantial morbidity, which mostly includes susceptibility to fractures and ischemic ulceration leading to lower-limb amputations (21).

One of the most distressing complaints of patients with diabetic neuropathy is pain. Painful DSPN is a common variant of DSPN, with characteristic features of neuropathic pain. The International Association for the Study of Pain (IASP) defines neuropathic pain as “pain caused by a lesion or disease of the somatosensory system” (22). Its pathophysiology is not fully understood. It is still unknown why some sub-groups of patients with the same disease develop neuropathic pain; even in this sub-group, the severity and impact of pain in individuals with the same conditions (23) are variable and unpredictable. A plausible explanation for this variation in neuropathic pain prevalence and severity is a complex interaction among genetic, psychosocial, and clinical risk factors in a more susceptible patient (24, 25). The pain can either be spontaneous or be provoked by painless (allodynia) or painful (hyperalgesia) stimuli. Pain is typically more severe during the night, which interferes with sleep and limits daily activities, and thus has a significantly negative impact on quality of life (19, 26). In a UK study, the prevalence of painful DSPN was reported to be 16.2% in DM1 patients (9). Age and diabetes duration were seen to be directly related to painful symptoms of neuropathy, with a slightly higher prevalence in females (38%).

The Michigan Neuropathy Screening Instrument (MNSI) was developed specifically to evaluate DSPN (27). This instrument has two complementary sections: section A refers to clinical history, and section B refers to the physical examination (foot appearance, ankle reflexes, vibration threshold, and 10-g Semmes-Weinstein monofilament test). Different questionnaires can been used to distinguish between neuropathic pain and other types of pain, such as the “Douleur neuropathique 4” (DN4), painDETECT, and Leeds Assessment of Neuropathic Symptoms and Signs (LANSS) (28). These scales are easy to apply in both clinical and epidemiological studies.

The aims of our study were to evaluate (1) the prevalence of DSPN and painful DSPN among patients with DM1 who are older than 18 years and (2) the determinant factors of neuropathy and pain in those patients.

## Materials and Methods

An epidemiological, cross-sectional, observational study that included patients from the catchment area of our hospital was performed. The study was approved by the local ethics commission [Ethics Committee of the Health Regional Administration of the North and Ethics Committee of Centro Hospitalar Universitário de S. João (CHUSJ)] and was carried out from April 2016 to August 2017, in accordance with the 1964 Principles of the Declaration of Helsinki and its later amendments.

### Participants

**Figure 1:**
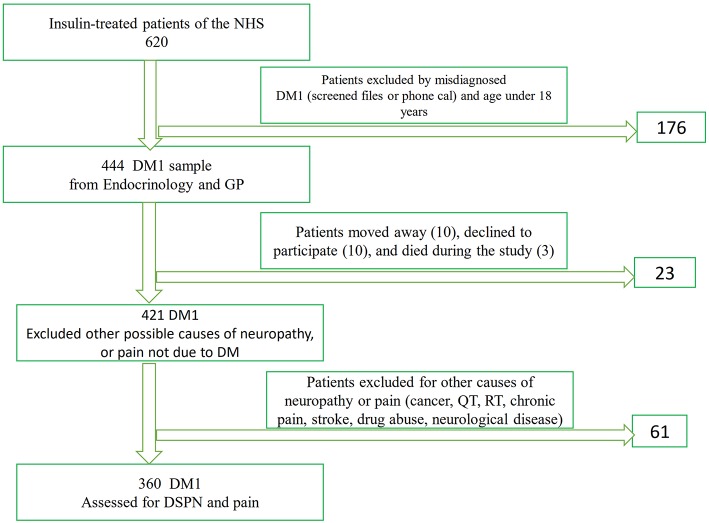
Flowchart of selection of the study participants with diabetes mellitus for the evaluation of neuropathy and pain. NHS, National Institute of Health; GP, general practitioner; DM, Diabetes Mellitus; DSPN, distal symmetrical sensory motor polyneuropathy; QT, Chemotherapy; RT, Radiotherapy.

A total of 330,386 inhabitants of the primary reference area of the CHUSJ who were registered at the health centers of Maia-Valongo and Eastern Porto were evaluated for the presence of DM and insulin treatment. We found 620 insulin-treated patients older than 18 years (Figure 1), some of whom were followed up at our hospital's department of endocrinology, whereas others were followed by their general practitioner, or at another hospital. We carefully evaluated all patients' files, and when some cases needed verification, these patients were either evaluated in person at the department of endocrinology or contacted by phone. A total of 444 of these patients both fulfilled the criteria for DM1 according to the American Diabetes Association (ADA), and satisfied the inclusion criteria. As shown in Figure 1, a flowchart of selection of the study participants with diabetes mellitus type 1 who were older than 18 years and after applying the exclusion criteria, we obtained a sample of 360 patients to evaluate the presence of neuropathy and pain.

The following exclusion criteria were used to evaluate the prevalence of DSPN and pain: patients with previous history of cancer or severe liver disease; patients who had undergone chemotherapy (QT) or radiotherapy (RT) or who required dialysis; patients with infectious diseases; patients with a history of alcoholism (more than 15 units per day) and/or drug abuse; patients with peripheral vasculitis, autoimmune diseases, cerebrovascular diseases with neurological sequelae, or chronic pain not due to DM; patients with a mental condition that could jeopardize the clinical symptom evaluation; and patients who refused to participate in the study or died during the course of the study. The use of anticonvulsants, antidepressants, opioids, or other medications for painful diabetic neuropathy was also considered to be an exclusion criterion.

Accordingly, we performed face-to-face interviews for the 360 DM1 patients, using a short version of a questionnaire for sociodemographic data, neuropathy, and pain.

### Instruments

We carried out a brief survey to obtain the socio-demographic data, body mass index, education (number of completed schooling years), working status, smoking, and alcoholism status (based on patient self-reporting), and date of diagnosis of diabetes. During the assessment, the subjects reported their medical and drug history. Blood pressure was measured at the time of the interview. We also reviewed the participants' clinical files to obtain information regarding cardiovascular risks factors and comorbidities, as well as biochemical parameters and HbA1c. Dyslipidemia was defined as follows: low density lipoprotein (LDL) cholesterol ≥160 mg/dL, triglycerides ≥200 mg/dL, high density lipoprotein (HDL) cholesterol <40 mg/dL, or use of lipid-lowering drugs. The data on diabetic complications including macrovascular complications (coronary, cerebrovascular, and peripheral arterial disease) as well as microvascular complications (nephropathy, retinopathy, and neuropathy) were obtained.

An increased AER of ≥20 mg/L (or 30 mg/24 h) and AER/creatinine ratio ≥30 mg/g creatinine in two urinary samples during the last 3 months were indicative of a diagnosis of nephropathy.

### Assessment of DSPN

DSPN diagnosis is mostly clinical, using suggestive clinical history and a neurologic examination. The Toronto Consensus criteria defines probable neuropathy as the presence of two or more of the following: neuropathic symptoms, decreased or absent ankle reflexes, or decreased distal sensation (2).

All patients underwent a neurological examination by a doctor, and the MNSI was used to evaluate neuropathy (2, 25). We used both sections A and B, as mentioned above. The MNSI Portuguese survey (29) can reliably screen diabetic neuropathy, with a cut-off value ≥ 3 (out of 12) in section A, and ≥2 (out of 10) in section B. According to our cut-off values of this scale, we defined the following: MNSI values ≤5 = no neuropathy, 6–10 = mild neuropathy, 11–15 = moderate neuropathy, and >16–22 = severe neuropathy.

A physical foot examination was conducted on all participants by the same doctor, who was blinded to the presence of neuropathy or pain.

### Pain Evaluation

All patients were asked whether they had chronic pain in their lower limbs and whether they experienced the pain daily for a period of at least 3 months (22). Pain intensity was evaluated using both the numeric rating scale (NRS) and the characteristics of nociceptive vs. neuropathic pain using the DN4 and LANSS scales. We considered neuropathic pain to be present if both scale scores were diagnostic, as described below.

If the answer was affirmative for the presence of pain, then its intensity was estimated using the NRS. Neuropathic characteristics of pain (30), such as pins and needles, crawling ants, numbness, freezing cold, and allodynia were all evaluated using the DN4 and LANSS scales (18).

The DN4 survey consists of four questions (including seven symptom-related sub- questions) and three physical examination signs (out of a total of 10 items). Patients with a score ≥4 were considered to have neuropathic pain (2).

The LANSS scale is quick and easily to administer, with five “yes or no” questions about symptoms as well as touch allodynia and altered pinprick threshold. In the Portuguese version of the scale, a cut-off score of ≥12 clearly suggests neuropathic pain (22).

### Statistical Analysis

Numerical variables were summarized by mean and standard deviation (SD), and categorical variables were summarized by proportions. In turn, proportion differences were compared using the Chi-square test, and mean differences were compared using a one-way ANOVA or Kruskal–Wallis test. The Pearson correlation coefficient was also calculated.

Associations between exposures (patients' characteristics) and the outcome (painful or painless DSPN) were estimated using multinomial logistic regression models to obtain crude and adjusted odds ratios (OR) and their respective 95% confidence intervals.

**Figure 2:**
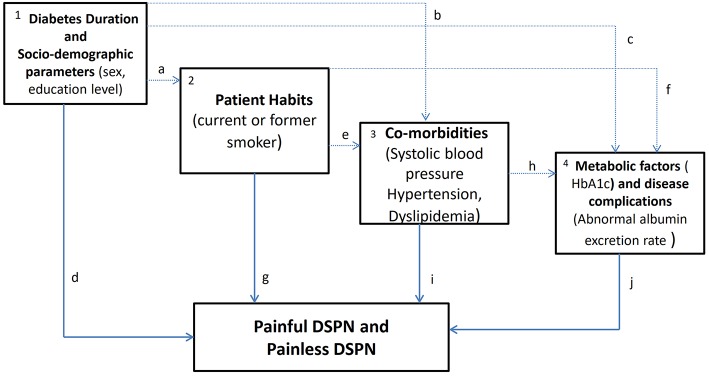
Theoretical framework of diabetes mellitus type 1 (DM1) determinants of neuropathy and pain. The variables within each block according to four conceptual levels were: (1) socio-demographic factors and diabetes duration, (2) patient habits, (3) co-morbidities and (4) metabolic factors and disease complications in DM1 patients with neuropathy and pain.

To better understand the overall risk factors and the “indirect or direct” determinant factors of the presence of neuropathy and pain, we based the analysis on a framework (31) that considers feasible pathways among the several risk factors. A step-by-step approach was used: first, a univariate model was fitted; second conceptually predefined blocks of variables were fitted separately (variables within each block mutually adjusted); and third, blocks including significant variables were introduced cumulatively into the analysis, using a fixed order based on a predefined theoretical framework (Figure 2). Figure 2 shows a theoretical framework of DM1 determinants of neuropathy and pain. Arrows represent theoretical causal relationships between determinants of painful and painless DSPN. Dashed gray lines represent possible indirect effects in the pathway between levels of determinants. Solid black lines represent the direct effects of factors, after adjustment for determinants in preceding levels that are not mediated by subsequent ones but may be explained by other factors (unknown or unmeasured).

(1) Diabetes duration and socio-demographic—economic factors may exert an effect on patients' habits (a), through its influence on subsequent co-morbidities (b), through metabolic factors/disease progression (c), and/or through unknown or unmeasured determinants (d). (2) Patient habits may affect painful or painless DSPN through subsequent co-morbidities (e), through metabolic factors (f), and/or through unknown or unmeasured factors (g). (3) Co-morbidities may influence painful or painless DSPN through metabolic factors (h), and/or through unknown or unmeasured determinants (i). (4) In this conceptual framework, metabolic factors and/or other unknown or unmeasured factors (j) would then influence painful or painless DSPN. In this study, we are particularly interested in the overall effects and direct effects (highlighted in bold, d, g, i, and j).

All variables with *p*-values lower than 0.1 were included in the final models. A significance level of 5% was set in all the analyses. The Statistical Package for Social Sciences (IBM SPSS Statistics for Windows, Version 23.0, 2011, Armonk) was used.

## Results

A total of 444 patients with DM1 who were older than 18 years were identified in a population of 330,386 inhabitants, which established a prevalence rate of DM1 of 0.15% (95% CI: 0.14–0.17). After the application of the exclusion criteria, a sample of 360 patients remained; the prevalence of DSPN and painful DSPN were evaluated in these patients. The characteristics of the participants recruited for the study are described in Table 1. Diabetes duration was 19.2 (± 12.47) years and the average HbA1c was 8 ± 1.75%. The presence of nephropathy (41.7%), retinopathy (45.5%), and neuropathy (42.8%) was similar in our sample. DSPN was present in 42.8% of participants (95% CI: 37.6–48.0) and painful DSPN was found in 18.9% of participants (95% CI: 15.0–23.4).

**Table 1:** Type 1 DM patients' characteristics after the application of the exclusion criteria of other cause of neuropathy and pain (*n* = 360).

**Features**	**Mean (SD) or *n* (%)**
**Age (years), mean (SD)**	42 (14.4)
**Sex**, ***n*** **(%)**	
Male	178 (49.4)
Female	182 (50.6)
**Ethnicity**, ***n*** **(%)**	
Caucasian	349 (96.9)
Black	10 (2.8)
Other	1 (0.3)
**Marital status**, ***n*** **(%)**	
Single	149 (41.4)
Married/living together	196 (54.4)
Separated/divorced	2 (0.6)
Widowed	13 (3.6)
**Referral** ***n*** **(%)**	
Followed up by a GP	105 (29.2)
Hospital	255 (70.8)
**Educational level (years), mean (SD)**	11.6 (4.4)
**Smoking habits**, ^*^n **(%)**	
Never smoked	220 (61.3)
Current smoker	99 (27.6)
Ex-smoker	40 (11.1)
**Alcohol**, ^*^n **(%)**	
Yes	139 (38.8)
No	219 (61.2)
**Diabetes duration (years), mean (SD)**	19.2 (±12.47)
**HbA1c %, mean (SD)**	8.1 (1.75)
**Body mass index mean (SD)**	24.9 (3.9)
**Blood pressure (mmHg)**	
Systolic, mean (SD)	131.9 (±15.1)
Diastolic, mean (SD)	72.3 (±29.9)
**Nephropathy**, ***n*** **(%)**	
Yes	150 (41.7)
**Retinopathy**, ***n*** **(%)**	
Yes	160 (45.5)
**Clinical DSPN**, ***n*** **(%)**	
Yes	152 (42.8)
**Painful DSPN**, ***n*** **(%)**	
Yes	67(18.9)

SD, standard deviation; GP, General Practitioner; HbA1c, glycated hemoglobin; DSPN, distal symmetrical sensory-motor polyneuropathy;

* *Some subjects did not report their smoking habits (1 = missing) and alcohol consumption habits (n = 2 missing)*.

Table 2 shows the descriptive statistics for the potential risk factors reported for painful DSPN, painless DSPN, no DSPN, and no pain, together with *p*-values that summarize the univariate association of each determinant factor with either painful DSPN, painless DSPN, no DSPN, and no pain.

**Table 2:** Comparison of the patients characteristics divided according to presence of neuropathy and pain.

**Characteristics**	**Total (*n*; SD)**	**Painful DSPN**	**Painless DSPN**	**No DSPN no pain**	***p*- value**
**Age (years), mean (SD)**	41.98 (±14.38)	49.87 (±15.73)	46.06 (±13.61)	37.67 (±12.58)	<0.0001^*^
**Diabetes duration (years), mean (SD)**	19.19 (±12.47)	29 (±13.77)	22.92 (±11.9)	14.42 (±9.58)	<0.001^*^
**Educational level, mean (SD)**	11.6 (±4.41)	9.72 (±4.35)	10.8 (±4.76)	12.5 (±4.02)	<0.0001^*^
**Sex**, ***n*** **(%)**					
Male	178 (49.4)	28 (41.2)	38 (44.2)	112 (54.4)	
Female	182 (50.6)	40 (58.8)	48 (55.8)	94 (45.6)	0.090
**Alcoholic habits**					
Yes	139 (38.8)	26 (18.7)	35 (25.2)	78 (56.1)	
No	219 (61.2)	42 (61.8)	51 (59,3)	126 (61.8)	0.920
**Smoker**, ***n*****(%)**					
Yes	139 (38.7)	22 (32.4)	40 (46.5)	77 (37.6)	
No	220 (61.3)	46 (67.6)	46 (53.5)	128 (62.4)	0.176
**SBP, mmHg; mean, SD**	131.9 (±15.1)	134.64 (±17.3)	133.51 (±14.2)	130.35 (±14.5)	0.069
**DBP, mmHg; mean, SD**	72.3 (±29.9)	71.03 (±13)	76.66 (±57.0)	70.94 (±11.7)	0.307
**Hypertension** ***n*** **(%)**					
Yes	80 (22.4)	26 (38,2)	29 (34.5)	25 (12.3)	
No	277(77.6)	42 (61.8)	55 (65.5)	180 (87.8)	**<0.001**
**Dyslipidemia**, ***n*** **(%)**					
Yes	74 (20.7)	16 (23.5)	25 (29.8)	33 (16.1)	
No	283 (79.3)	52 (76.5)	59 (70.2)	172 (83.9)	0.028
**HbA1c, mean (SD)**	8.13 (±1.75)	8.285 (±1.52)	8.49 (±1.68)	7.93 (±1.83)	0.036
**Albuminuria**, ***n*** **(%)**					
Yes	88 (24.6)	32 (47.8)	33 (38.4)	23 (11.2)	
No	270 (75.4)	35 (52.2)	53 (61.6)	182 (88.8)	**<0.001**

*Values are presented as n (%), or as mean ± standard deviation. P <0.05, SBP, systolic blood pressure; DBP, diastolic blood pressure; HbA1c, glycated hemoglobin*.

* *ANOVA. In bold were indicated the significant values*.

The subgroup of DM1 patients with no DSPN and no pain tended to be younger than those with painless DSPN. The painless DSPN group had an intermediate mean age, whereas the painful DSPN group had the highest mean age (*p* < 0.001) (Table 2). A similar trend was also found for diabetes duration in the three groups of patients (*p* < 0.001). For the parameter of educational level, we found a negative association; diabetic patients with no DSPN and no pain have a higher level of education (12.5 years) compared to those with painless DSPN (10.8 years) and painful DSPN (11.6 years) *p* < 0.001.

We found no association between sex, alcohol consumption, history of smoking, systolic blood pressure (SBP), and diastolic blood pressure (DBP) and all groups of DM1 patients (with or without neuropathy or pain).

However, we did find an association between hypertension (*p* < 0.001), dyslipidemia (*p* < 0.028), HbA1c (*p* = 0.036), and albuminuria (*p* < 0.001) in all the patients, either with or without neuropathy or pain.

Table 3 shows the multivariable analysis of associations between neuropathy with or without pain and the determinant factors of our framework. Accordingly, the analysis was based on a framework with four conceptual levels: (1) diabetes duration and socio-demographic parameters, (2) patients' habits, (3) co-morbidities, and (4) metabolic factors and disease complications. The diabetes duration factor was positively associated with painful DSPN (OR = 1.107, 95% CI: 1.107–1.139) and painless DSPN (OR = 1.069, 95% CI: 1.043–1.096); education level was negatively associated with painful DSPN (OR = 0.889, 95% CI: 0.826–0.957); sex (female) was positively associated only with painless DSPN (OR = 1.769, 95% CI: 1.007–3.107); being a current or former smoker was positively associated only with painless DSPN (OR = 1.940, 95% CI: 1.069–3.518); hypertension was positively associated with painful DSPN (OR = 2.474, 95% CI: 1.110–5.512); and painless DSPN (OR = 2.565, 95% CI: 1.252–5.256). HbA1c was positively associated only with painless DSPN (OR = 1.193, 95% CI: 1.018–1.399). The education level effect was attenuated after the inclusion of co-morbidities and metabolic factors, while the sex effect was de-attenuated after adjusting for patients' habits in painless DSPN.

**Table 3:** Multivariate analysis of association with neuropathy with or without pain and risk factors.

	**Model 1 OR** **(95% IC)**	**Model 1**	**Model 2 OR** **(95% IC)**	**Model 2**	**Model 3**	**Model 3**	**Model 4**	**Model 4**
	**Painful** **DSPN**	**Painless** **DSPN**	**Painful** **DSPN**	**Painless** **DSPN**	**Painful** **DSPN**	**Painless** **DSPN**	**Painful** **DSPN**	**Painless** **DSPN**
Diabetes duration (years)	**1.107**	**1.069**	**1.107**	**1.070**	**1.104**	**1.062**	**1.100**	**1.058**
	**(1.077–1.139)**	**(1.043–1.096)**	**(1.077–1.138)**	**(1.044–1.097)**	**(1.072–1.136)**	**(1.035–1.090)**	**(1.065–1.135)**	**(1.028–1.088)**
Education level (years)	**0.889**	**0.920**	**0.893**	**0.920**	**0.906**	0.948	0.940	0.978
	**(0.826–0.957)**	**(0.863–0.980)**	**(0.830–0.961)**	**(0.863–0.981)**	**(0.837–0.981**)	(0.884–1.017)	(0.864–1.023)	(0.908–1.053)
Sex (female)	1.566	1.531	1.59	**1.769**	1.675	**2.091**	1.766	**2.142**
	(0.829–2.957)	(0.892–2.629)	(0.821–3.078)	**(1.007–3.107)**	(0.849–3.304)	**(1.154–3.788)**	(0.881–3.540)	**(1.171–3.917)**
Smoker			1.071	1.702	1.150	**1.940**	0.975	1.726
			(0.541–2.119)	(0.965–3.004)	(0.570–2.321)	**(1.069–3.518)**	(0.469–2.028)	(0.940–3.170)
Systolic BP, mmHg					1.021	1.015	1.018	1.014
					(0.999–1.043)	(0.996–1.034)	(0.996–1.040)	(0.995–1.033)
Hypertension					**2.474**	**2.565**	**2.666**	**2.716**
					**(1.110–5.512)**	**(1.252–5.256)**	**(1.168–6.087)**	**(1.299–5.681)**
Dyslipidemia					0.749 (0.322–1.739)	1.521 (0.756–3.058)	0.760 (0.946–4.773)	1.564 (0.763–3.203)
HbA1c							1.186 (0.975–1.442)	**1.193** **(1.018–1.399)**
Abnormal albumin excretion rate							2.125 (0.946–4.773)	1.952 (0.935–4.077)

*Values are presented using the following: Reference group = no DSPN and no pain; Model 1 = duration of diabetes, education level (years), and sex; Model 2 = Model 1 plus current or former smoker; Model 3 = Model 2 plus systolic blood pressure, hypertension, and dyslipidemia; Model 4 = Model 3 plus glycated hemoglobin (HbA1c) and abnormal albumin excretion rate for the last 3 months. In bold were indicated the significant values*.

## Discussion

In this study, we included all patients with DM1 after excluding possible confounders (*n* = 360) who resided in our reference area to establish the prevalence of diabetic neuropathy (painful DSPN and painless DSPN). The presence of DSPN in our sample was found to be 42.8% (95% CI: 37.6–48.0%). Previous epidemiological studies have yielded similar results, ranging from 22.7 to 54% (30, 32).

Our values are higher than those of other studies, such as a Spanish study (1) wherein the prevalence of DSPN was found to be 12.9%, and a UK study (9), wherein it was 22.7% (21.0–24.4%). One explanation for this difference could be that the disease duration was longer in our sample (19 years) compared with 13.8 years for the Spanish study, and 17 years for the UK Study. In addition, the patients in our study were younger at diagnosis (average age 30.8 ± 0.6 years) (data not shown) than the UK study (37.6 ± 12.9 years) but were similar to the Spanish study (30.5 ± 0.6 years).

As previously mentioned, nephropathy was present in 41.7%, retinopathy in 45.5%, and clinical neuropathy in 42.8% of patients. The most reasonable explanation for this diabetic triopathy could be the longer duration of diabetes in our patient sample, although there was only an association between albuminuria and neuropathy in our study.

Our study shows that diabetes duration has a direct effect on the development of painful and painless DSPN among DM1 patients, although the precise mechanisms remain unclear.

Education level has an indirect effect on the co-morbidities and the metabolic effects or disease progression in the development of painful and painless DSPN.

To the best of our knowledge, this association of education level and the presence of DSPN has not been observed in other studies, although Pirart et al. (33) did note that DSPN was twice as frequent among the poorest socio-economic groups. In our data, patients with painful DSPN were less educated than those in the other groups; the patients with DM1 without DSPN and no pain had a higher education level. In our country, this has a social impact because a higher education level might mean an access to other sources of information besides the physician and an awareness of DM1 related complications. However, we cannot extrapolate that being less educated is associated with the poorest socio-economic demographic, since we did not evaluate the familiar and socioeconomic levels using the Graffar and APGAR scales, respectively.

In our LANSS scale validation study for the Portuguese population, the patients included in the sample consulted in the same hospital used in the current study as well in another hospital in the north of Portugal. We evaluated the familiar function using the APGAR scale and found that in the neuropathic pain patients, the familiar function and the intensity of pain (measured using NRS) were significantly higher. Additionally, we found an inverse relationship between APGAR and NRS in the neuropathic pain group that could be explained by the better familiar support. In the current study, we found a correlation between the subgroup of patients with DM1 with lower education level and painful DSPN. Thus, even if we did not evaluate the relationship between APGAR and NRS in the current study, we believe that if we had evaluated these features, we would had found the same relationship as that in our previous study.

We observed that female sex was only positively associated with painless DSPN. However, in a UK study (34) a significantly great proportion of female patients reported painful neuropathy symptoms despite a lower frequency of clinical neuropathy. Even after adjustments for age and diabetes duration and differences in clinical neuropathy had been made, women still had a 50% increased risk of neuropathy (34). Few studies have reported a male predominance (2). In agreement with our results, some authors have demonstrated that women were more affected than men (31, 35), and Raputova et al. demonstrated that the proportion of women displaying severe neuropathy was higher compared with that of men (36). Some studies have shown no differences between sexes (1). It is hard to propose a clear explanation for these discrepancies, although different confounding factors could contribute to them. For instance, in our data, the effect of smoking (current or former) attenuates the sex effect on painless DSPN development.

Among the co-morbidities (systolic blood pressure, hypertension, and dyslipidemia), hypertension is the only variable with a positive association with painless and painful DSPN through a direct effect. As mentioned in other studies (13), hypertension and increased level of triglycerides have been shown to play an important role in the development of DSPN. There was a positive and significant association between HbA1c and painless DSPN, but not with painful DSPN, which leads us to the conclusion that the mechanisms of pain in diabetic patients is not clear.

Compared to the literature, the strength of our study is the high number of DM1 patients that were included without being confounded with type 2 diabetic patients. Another strength of our study was that we based our analysis on a predefined theoretical framework, wherein we defined potential determinants, and the prevalence of painful DSPN and painless DSPN was assessed using clinical history and a physical examination by a trained physician for all of the participants. As all the evaluations were performed by the same experiment physician we can exclude any differences between observers, what gives strength to our work. Another important strength of this study is its determination of the prevalence of DM1 in a large geographical area.

The standardized neurological evaluation using a validated questionnaire [MNSI (37) examination in this case] increased the recognition of neuropathy among patients with a lesion of large fibers (3). However, this prevalence could be underestimated, as this disease preferentially affects small fibers (38) during the early stage of its onset. As we studied patients older than 18 years who had the disease for more 19 years, our value of 42.8% of neuropathy would not vary much if other specific tests for small fibers such as quantitative sensory testing (19, 39) were performed.

The use of two surveys [LANSS and DN4 (40, 41)] as screening tools for neuropathic pain is a strength of this study, as the majority of epidemiological studies (35, 42) only use the DN4 scale. Both surveys are well-validated for our population, and have the advantage of being specific, sensitive, and easy to administer.

Our study does have certain limitations. For example, the study was carried out in only one center; however, we observed all patients with DM1 in the catchment area of CHUSJ. Thus, the dataset was large as it included both hospital- and primary care-followed patients. Our results suggest that the majority of DM1 patients are followed by an endocrinologist (data not shown), which could be one reason that we did not find a high incidence of major amputation in our results. The use of more specific instruments, such as quantitative sensory testing (43, 44), skin biopsy (45), or corneal confocal microscopy (46–48) for DM1 cases could provide information about more specific mechanisms of painful neuropathy.

In conclusion, the prevalence of DM1 was found to be relatively low (0.15%) in our region of North Portugal compared to that in northern Europe. The incidence of DSPN and painful DSPN are higher in our study than that in previous studies. The most important determinants were diabetes duration for painful neuropathy and sex (male) as well as HbA1c for painless neuropathy. In addition, diabetes duration, sex, hypertension, and HbA1c directly increased the odds of occurrence of neuropathy and pain—although sex (female) and HbA1c only had a direct effect on the development of painless neuropathy. Education level had an indirect effect on the development of neuropathy, both with or without pain.

Future research should analyze in more depth the possible mechanisms of pain in DM1 patients. Furthermore, the determinants that have a higher impact on the development of painful neuropathy and painless neuropathy among these patients should be identified.

## Data Availability

All datasets generated for this study are included in the manuscript and/or the supplementary files.

## Ethics Statement

The study was approved by the local ethics commission (Ethics Committee of the Regional Administration of the North (RAN) and Ethics Committee of CHSJ) and was carried out from April 2016 until August 2017, in accordance with the 1964 Principles of the Helsinki Declaration and its later amendments.

## Author Contributions

MB conceived and designed the idea, did data collection, wrote and drafted the manuscript. AS, SO, and LR did data collection. FR did literature review. CM and RS reviewed the manuscript. MS performed the data analysis. DC designed, contributed to the reviewing of the final manuscript. All authors approved the final format of the submitted manuscript.

### Conflict of Interest Statement

The authors declare that the research was conducted in the absence of any commercial or financial relationships that could be construed as a potential conflict of interest.
